# Immunopathogenesis of Behcet's Disease

**DOI:** 10.3389/fimmu.2019.00665

**Published:** 2019-03-29

**Authors:** Bainan Tong, Xiaoli Liu, Jun Xiao, Guanfang Su

**Affiliations:** Department of Ophthalmology, The Second Hospital of Jilin University, Changchun, China

**Keywords:** Behcet's disease, pathogenesis, cytokines, autoimmune diseases, biotherapy/target therapies

## Abstract

Behcet's disease (BD) is a chronic systemic inflammatory vasculitis of unknown etiology characterized by recurrent episodes of oral aphthous ulcers, genital ulcers, skin lesions, ocular lesions, and other manifestations. Although the pathogenesis of BD is unclear, some studies have shown that immunological aberrations play an important role in the development and progression of BD. Infection-related trigger factors, including antigens and autoantigens, are believed to mediate the development of BD in patients with a genetic predisposition and subsequently activate the innate and adaptive immune systems, resulting in the production of numerous cytokines and chemokines to combat the infection-related factors. The study of the immunological mechanism of BD paves the way for the development of innovative therapies. Recently, novel biotherapy approaches, including interferon-α (IFN-α), tumor necrosis factor-α (TNF-α) antagonists, and other agents that target interleukins and their receptors, have shown promising results in the treatment of patients with refractory BD and have improved the prognosis of BD. In this review, we provide the current concepts of BD immunopathogenesis.

## Introduction

Behcet's disease (BD) is a chronic recurrent multisystemic disease that involves oral aphthous ulcers, genital ulcers, skin lesions, ocular lesions, gastrointestinal and central nervous system (CNS) abnormalities, and other pathologies (1). The epidemiology of BD is uniquely distributed along the ancient Silk Road from Mediterranean countries, including Turkey (370 cases per 100,000 population), to Middle Eastern and East Asian countries, but BD is rarely encountered in Northern Europe (0.64 cases per 100,000 population), North America (0.12–0.33 cases per 100,000 population), Australia, and Africa (2, 3). The pathogenesis of BD is not completely clear, but genetic susceptibility, trigger factors, and immunological abnormalities have been reported to play a decisive role in BD development (3).

BD is closely related to the presence of the HLA-B^*^51 allele of the major histocompatibility complex, which may play a role in BD pathogenesis via a combination of different HLA class I-associated functions and/or structural characteristics of the HLA-B^*^51 heavy chain (4). A meta-analysis using data from 78 independent studies, which investigated 4,800 BD patients and 16,289 controls from around the world, showed that the odds ratio (OR) of having the HLA-B5/B^*^51 alleles for developing BD was 5.78 (95% confidence interval (CI) 5.00–6.67) (5). Although HLA-B^*^51 is the known genetic factor most closely associated with BD, it accounts for <20% of the genetic risk. A genome-wide association study (GWAS) and meta-analysis identified common variants in interleukin 10 (IL-10) and at the IL-23R–IL-12RB2 locus that predispose individuals to BD. Expression studies have shown that the disease-associated IL-10 variants are associated with reduced expression of this anti-inflammatory cytokine, which may lead to a susceptible inflammatory state, thus increasing susceptibility to BD (6).

However, genetic factors cannot fully explain the pathogenesis of BD. The environmental trigger hypothesis has also been proposed in BD patients with genetic susceptibility. Trigger factors such as bacteria or viruses may have a high affinity for HLA-B51 molecules (4). In the environmental trigger hypothesis for BD with genetic susceptibility, BD is manifested by the involvement of the innate immune system, which is sustained by the adaptive immune response of T cells to infectious antigens or autoantigens (2). In addition, immune-mediated networks play a role in the inflammatory cascade. Recently, the pathogenesis of BD has been classified as the intersection of autoimmune and autoinflammatory syndromes (3). On the one hand, observations suggest that BD may have inflammatory properties. The definitions of autoinflammatory diseases describe numerous seemingly unprovoked recurrent inflammatory episodes caused by perturbation of cytokine networks (7). Remission and exacerbation of recurrent episodes are observed not only in BD but also in other autoinflammatory diseases. Unlike other autoimmune diseases, the pathogenesis of BD has not been found to be associated with specific autoantibodies, and BD is related to certain autoinflammatory diseases. However, evidence also indicates that BD has the characteristics of autoimmunity. Similar to other autoimmune diseases, BD is associated with class I MHC (HLA-B51). Activation of the adaptive immune system in autoimmune diseases is the main cause of inflammatory processes. Th1 and Th17 cells play an important role in the pathogenesis of BD (8). Therefore, characterization of BD as an autoinflammatory or autoimmune disorder is complicated. Recently, the immunopathogenesis of BD has been extensively studied, and various immune cells and cytokines have been found to be involved in the pathogenesis of BD (9, 10). In this paper, we review advances in the knowledge regarding the immunopathogenesis of BD in terms of antigens, innate immune cells, adaptive immune cells and cytokines.

## The Role of Antigens in the Pathogenesis of Behcet's Disease

The environmental trigger hypothesis of BD development in patients with genetic susceptibility was proposed many years ago. The trigger factors include bacterial infections, viral infections and the presence of abnormal autoantigens. Major bacteria, such as *Streptococcus sanguis, Helicobacter pylori*, and *Mycoplasma*, and viral infections, including herpes simplex virus 1, Epstein-Barr virus, hepatitis, and cytomegalovirus, have been investigated in the etiology of BD (3). The most common microorganism isolated from BD patients is *Streptococcus*. Clinical observations have uncovered a relationship between streptococcal infection and BD, similar to the relationship observed between tonsillitis and dental caries, which occur together at a high incidence. Dental treatment was shown to aggravate BD, and antibacterial treatment had a beneficial effect on mucocutaneous and arthritis symptoms (11). *Staphylococcus aureus* and oral *Streptococcus* have been identified in the skin lesions of patients with BD (12). Studies have provided serological evidence that chronic *C. pneumoniae* infection was associated with Behcet's disease. Ayaslioglu et al. found that patients with BD have higher IgA seropositivity to *Chlamydia pneumoniae* (13). Although some microbial infections are believed to be trigger factors of BD, there is no evidence that BD is the result of direct infection by viruses and bacteria (10). Studies have shown that autoantigens, via molecular mimicry, play a key role in the development of BD. Several autoantigens have been observed, including the heat-shock protein (HSP) 60 kDa and HSP70 kDa proteins, S antigen, interphotoreceptor retinoid-binding protein (IRBP), α-tropomyosin, and αβ-crystallin (3).

Molecular mimicry based on sequence homology between microbial and human HSP peptides triggers autoimmune responses in patients with BD (14–16). Through the study of the sequence homology between streptococcal cell wall M proteins and tropomyosin, shared immunological epitopes were revealed. The similarity between this streptococcal surface protein and tropomyosin suggests that molecular mimicry may lead to the inflammation seen in BD via the induction of an immunoreaction to tropomyosin (17). Indeed, an immunoreaction to S antigen and IRBP, which are retina-specific autoantigens, has been suggested to be involved in the pathogenesis of BD. The non-cross-reactive immune proteins S antigen and IRBP induced experimental autoimmune uveitis with similar pathology in different rodent models (18). This study showed that in both BD patients and healthy controls, cytokines associated with T helper Th1- and Th17-mediated immune responses were produced by peripheral blood mononuclear cells (PBMCs) stimulated with S antigen or IRBP. The levels of IL-6 produced by S antigen-specific T cells and the levels of IL-6, IFN-γ, and IL-17 produced by IRBP-specific T cells in BD patients were significantly higher than those in healthy controls (19, 20).

## The Role of Innate Immune Cells in the Pathogenesis of Behcet's Disease (Figure 1)

### Natural Killer (NK) Cells

NK cells are the main components of innate immunity. NK cells not only play a cytotoxic role in infected cells and tumor cells but also regulate the function of other immune cells, including dendritic cells (DCs) and T cells, by secreting cytokines (21). Based on the surface density of the CD56 molecules expressed by human NK cells, NK cells can be divided into two subsets: CD56^bright^CD16^−^ and CD56^dim^CD16^+^ NK cells. CD56^dim^CD16^+^ NK cells account for approximately 90% of human peripheral blood NK cells; cells in this subset are characterized by high cytotoxicity to tumor cells and virus-infected cells but a very low ability for cytokine production. In contrast, CD56^bright^CD16^−^ NK cells are considered to be regulatory NK cells due to their low cytotoxicity, but these cells produce high levels of cytokines during stimulation (22). These cells can produce large amounts of IFN-γ, granulocyte-macrophage colony-stimulating factor (GM-CSF) and chemokines, although cell proliferation is not active in response to IL-2 (23). NK cell activation is controlled by signals from activating and inhibitory receptors. NK cell surface inhibitory receptors recognize self MHC class I molecules and transduce inhibitory signals to protect the body from NK cells in states of health. Under conditions of pathogen infection, inflammation, tumor development or injury, the expression of self MHC class I molecules on the NK cell surface is downregulated, which leads to a decrease in inhibitory signaling and the subsequent activation of NK cells (24). In autoimmune diseases, the frequency of NK cells is decreased and their cytotoxicity is impaired, suggesting that NK cells play a protective role in controlling autoimmunity (22). Hasan et al. reported that the number of NK cells in the peripheral blood of BD patients was significantly decreased, a change that was related to disease activity. In addition, that study showed that CD56^bright^CD16^−^ and CD56^dim^CD16^+^ NK cells were depleted in the peripheral blood of BD patients compared with the numbers of these cells in healthy controls (25). Peripheral blood depletion of NK cells in BD patients may reflect increased homing of these cytotoxic cells to inflammatory sites and activation and maintenance of tissue inflammation in BD patients through Th1 cytokine production, resulting in cytotoxicity in the active phase.

**Figure 1:**
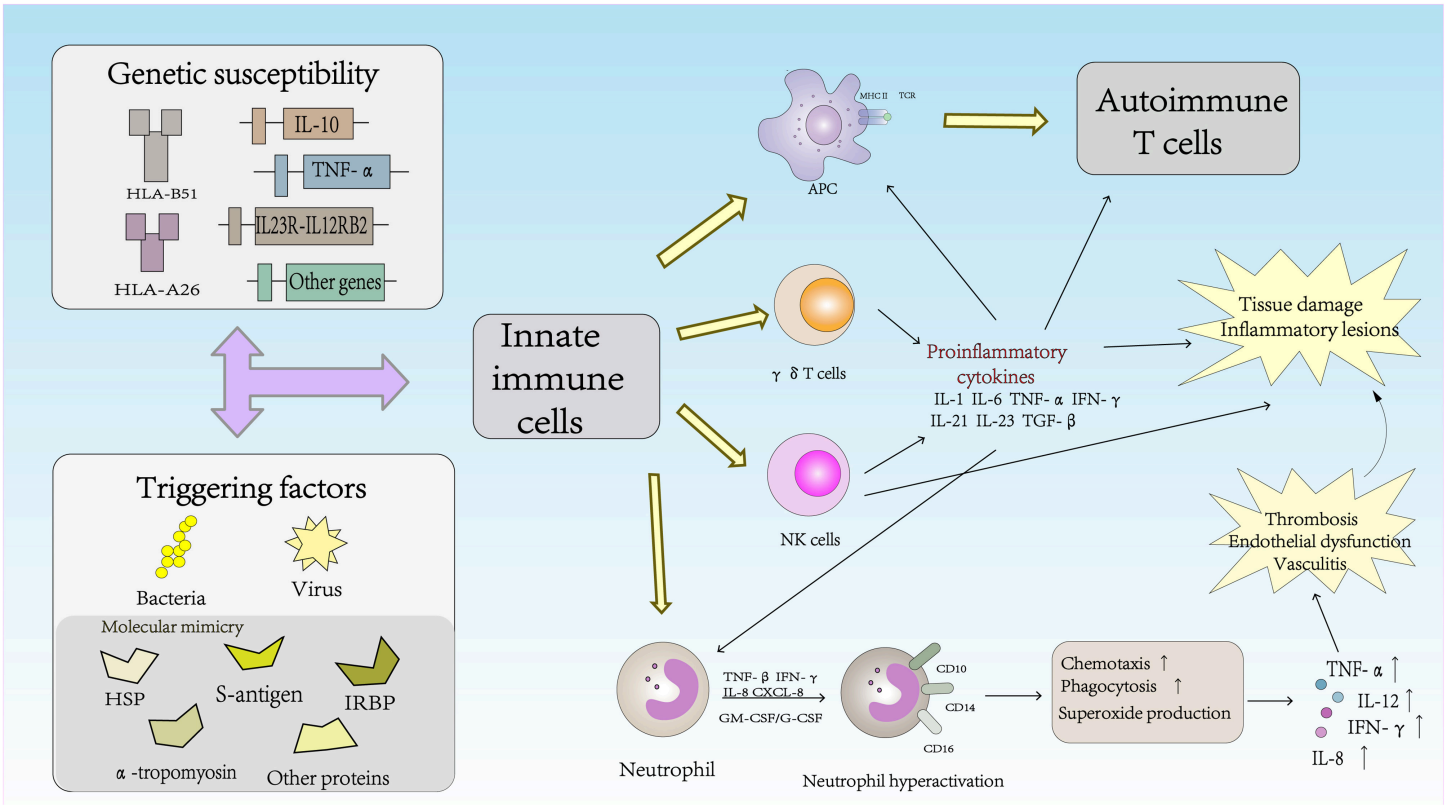
The role of innate immune cells in the pathogenesis of Behcet's disease.

NK cells can also be divided into the NK1, NK2, NK17, NKreg, and NK22 cell types according to the cytokines secreted (26). NK cells have recently been reported to play an important role in Th1 dominance in BD patients, and the Th1-type cytokine IFN-γ is known to inhibit Th17 cells from producing cytokine IL-17 (27). Cosan et al. reported an advantage of the NK1 subset, but compared with healthy subjects, the proportions of NK2, NK17, and IL-10-secreting cells in BD patients were lower, and similar cytokine profiles have been observed in BD patients with mucosal skin involvement (28). Because of the inhibitory effect of IFN-γ, the dominant function of NK1 cells was increased, and increased secretion of IFN-γ may inhibit NK2, NK17, and NKreg cells in BD patients. The NK1/NK2 paradigm has been shown to control pathogenic Th1- or Th2-biased responses. The study suggested that NK cells play an active role in the remission of BD patients through NK2 polarization. NK cells may regulate the Th1 response mediated by NK2 cells to control disease remission in patients with BD (29).

### *γδ* T Cells

γδ T cells play an important role in the regulation of the autoimmune response. In adult peripheral blood, γδ T cells accounted for 0.5–5% of the total PBMC lymphocytes. Similar frequencies of γδ T cells were found in the thymus, lymph nodes, tonsils and gut- and skin-associated lymphoid systems (30). Although γδ T cells usually represent very of the total lymphocytes in peripheral lymphoid organs under normal conditions, the number of γδ T cells can increase in just a few days to represent more than 50% of all circulating T cells during infection (31). TCRVγ9Vδ2^+^ T cells, the major subset of γδ T cells in the peripheral blood, can produce multiple proinflammatory cytokines in the presence of growth factors and cytokines. Recent studies have shown that γδ T cells play an important role in the inflammatory lesions associated with experimental models of autoimmune diseases. Activated γδ T cells promote the activation of IL-17^+^ uveitogenic αβ T cells and accelerate the development of experimental autoimmune uveitis (EAU) (32). Sutton et al. demonstrated a new natural mechanism in which γδ T cells activated by IL-1β and IL-23 are important sources of innate IL-17 and IL-21 production and IL-1 and IL-23 may mediate autoimmune inflammatory diseases (33). Furthermore, the activity and high proliferative response of γδ T cells to different microbial infections have been reported in BD patients. TCRVγ9Vδ2^+^ can be stimulated by isopentenyl pyrophosphate (IPP), a mycobacterial antigen. IPP-specific TCRVγ9Vδ2^+^ Th1-like cells are generated from intraocular fluid in patients with ocular uveitis due to BD (34, 35). γδ T cells were associated with active BD and higher CD69 expression and IFN-γ and TNF-α production. The changes in γδ T cells in BD patients indicated that γδ T cells were regulated, which altered their activity. Parlakgul et al. found no increase in γδ T cells in active BD patients in a recent study. However, functional changes in these cells were implicated according to the surface receptor on γδ T cells. The cytokine response of γδ T cells was decreased, which resulted in weakened regulation of these cells in BD patients (36, 37).

### Neutrophil Cells

Neutrophils play a vital role in the innate immune response and are the first line of defense against infectious diseases. Neutrophils can damage host cells and tissues while destroying microbes. Therefore, tissue damage is one of the main triggers of inflammation, which in turn triggers the immune response (38). Cell surface antigens such as CD10, CD14, and CD16 are expressed in neutrophils and are related to neutrophil function. Neutrophils in BD patients exhibit high intrinsic activation that may be associated with HLA-B^*^51 and are usually involved in perivascular infiltration in BD lesions (39). Hyperactive neutrophils can increase chemotaxis, phagocytosis, and superoxide production (2). The production of reactive oxygen species (ROS) is a normal characteristic of neutrophils. Neutrophil-mediated oxidative stress abnormalities may play an important role in the pathogenesis of BD, and advanced oxidation protein products (AOPPs), may be a useful marker for monitoring the progression and severity of disease activity in patients with BD (40). High levels of proinflammatory cytokines and chemokines, including IL-8, TNF-α, INF-γ, granulocyte-macrophage colony-stimulating factor (GM-CSF)/G-CSF, and CXCL-8, may be associated with the primary status of neutrophils (41, 42). BD, also known as chronic vasculitis, is characterized by venous thrombosis, aneurysms and occlusions. Unlike classic vasculitis, pathological studies in BD patients have shown a lack of true necrotizing vasculitis, granuloma, or immunocomplex deposition (41). Histopathological analysis has shown that arteries and veins are infiltrated by neutrophils and lymphocytes, which results in vascular endothelial dysfunction (43). Thrombosis occurs in approximately 25% of all BD patients, and the incidence of thrombosis in veins is higher than that in arterial vessels. Endothelial dysfunction and neutrophil vascular inflammation are key factors mediating thrombosis in patients with BD (3). Becatti et al. emphasized that neutrophil activation promoted fibrinogen oxidation and thrombosis formation in BD. In particular, their results suggest that an altered fibrinogen structure and impaired fibrinogen function are related to neutrophil activation and the production of enhanced ROS, which are mainly derived from neutrophil NADPH oxidases (44).

## The Role of Autoimmune T Cells and Cytokines in the Pathogenesis of Behcet's Disease (Figure 2)

### Th1 Cells and Cytokines in Behcet's Disease

The Th1 immune response plays an important role in the pathogenesis of BD. The expression levels of Th1 cells and related cytokines are associated with the activity of BD. Studies have found that the frequencies of Th1 cells and their cytokines and transcription factor T-bet were significantly higher in patients with active BD than those in patients with inactive BD. Increased levels of Th1 cytokines, such as IL-2, IL-12, IL-18, and IFN-γ, have been reported in the PBMCs of patients with active BD (45, 46).

**Figure 2:**
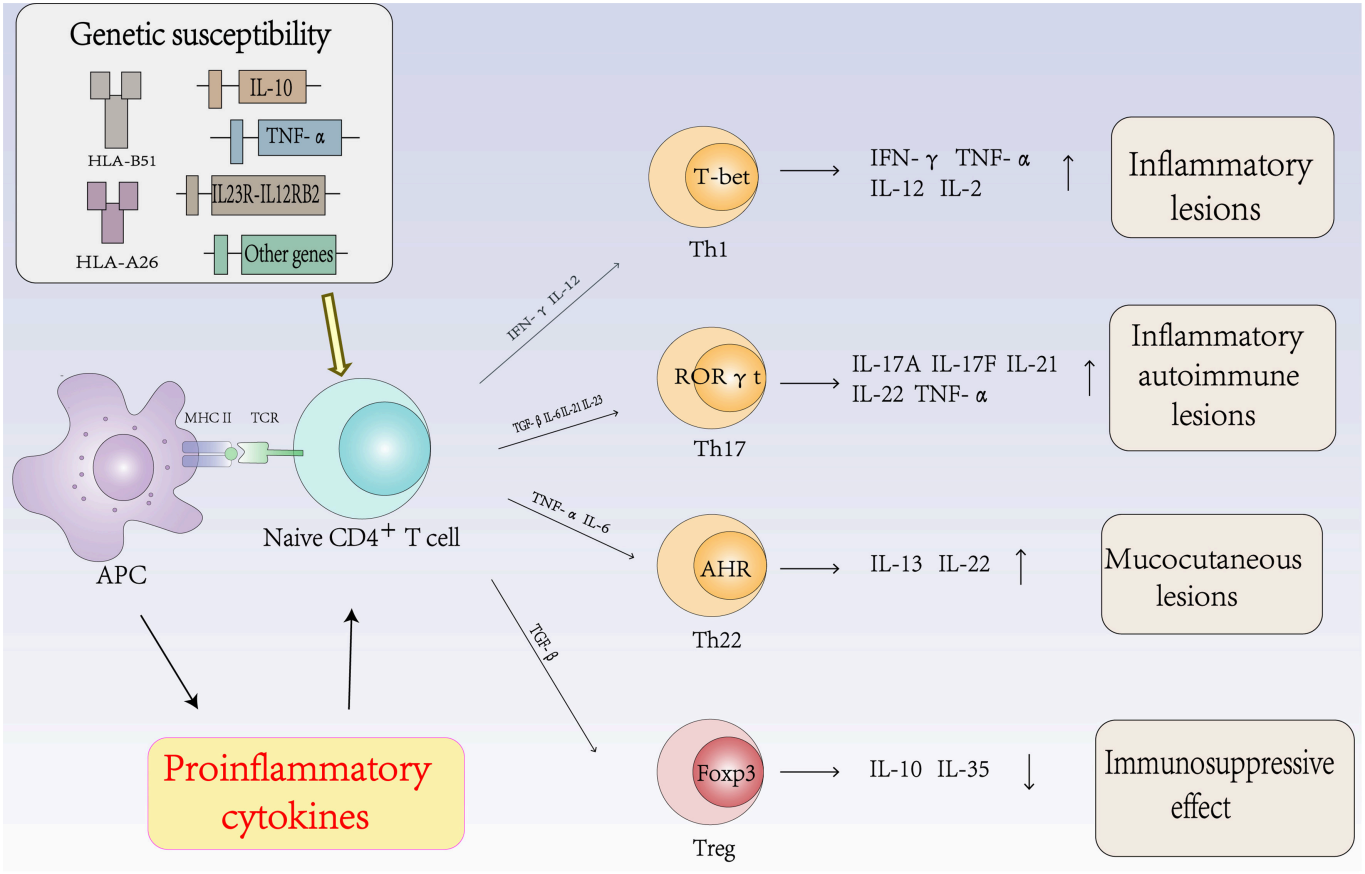
The role of autoimmune T cells and cytokines in the pathogenesis of Behcet's disease.

IL-12 and IFN-γ are the signature cytokines of the Th1 lineage. IL-12, which is composed of 2 heterodimeric subunits (p35 and p40), is produced by DCs, macrophages and B cells and is a key Th1-inducing cytokine (47). Th1 cells that produce IFN-γ activate macrophages, which are responsible for cell-mediated immunity to intracellular pathogens and are associated with many organ-specific autoimmune diseases, including BD (48). Ahn et al. reported that the levels of IFN-γ in the aqueous humor of patients with BD were significantly higher than those in patients with uveitis caused by other diseases (46). In addition, El-Asrar et al. reported that the level of IFN-γ in the aqueous humor of BD patients was significantly higher than that in patients with Vogt-Koyanagi-Harada (VKH) disease and HLA-B27-associated uveitis (48).

IL-18 is a proinflammatory cytokine that plays an important role in the immune response of Th1 cells. The functions of IL-18 are to promote the production of IFN-γ by activating NK cells, to induce the cytotoxic activity of NK cells and to stimulate T cells to secrete IL-12 and IFN-γ (9). Oztas et al. reported that the levels of IL-18 and TNF-α were increased in the serum of patients with BD, which indirectly supports the hypothesis that these proinflammatory cytokines are related to the pathogenesis of BD (49). Furthermore, Musabak et al. showed that the serum level of IL-18 was significantly increased in all subgroups compared with that in the healthy control group and correlated with the disease activity score in patients with active disease. This study suggested that IL-18 is involved in the pathogenesis of BD and that its level is closely related to disease activity (50).

### Th17 Cells and Cytokines in Behcet's Disease

Although BD was once considered a Th1-mediated disease, Th17 cells are central in the process of autoimmune diseases. Accumulating evidence suggests that Th17 cells regulate inflammation and autoimmune diseases. Cytokines such as IL-6, TGF-β, IL-21, and IL-23 promote the differentiation of Th0 cells into Th17 cells by activating signal transducer and activator of transcription (STAT) 3 and various transcription factors. Th17 cells then produce cytokines such as IL-17A, IL-17F, IL-21, IL-22, and IL-23 to regulate inflammation and autoimmunity (51). The expression levels of Th17 cells and related cytokines are associated with the activity of BD. Chi et al. found that the frequencies of Th17 cells and their cytokines and transcription factor RORγt were significantly higher in active BD patients than those in inactive BD patients (52, 53). The frequency of circulating Th17 cells in patients with active BD has also been reported to be significantly higher than that in the same patients in the remission stage (54).

IL-23 is a member of the IL-12 family, sharing a p40 subunit with IL-12. IL-23 is involved in the pathogenesis of BD by promoting the production of IL-17 by Th17 cells. Studies have shown that polymorphisms of IL-23R were related to BD susceptibility. Jiang et al. reported that rs17375018 in the IL-23R gene had a strong correlation with BD uveitis in a Chinese Han population (55). Recent studies have also shown that IL-23 does not promote the development of IFN-γ-producing Th1 cells but is one of the essential factors in increasing a pathogenic CD4^+^ T cell population, which is characterized by the production of IL-17, IL-6, and TNF-α (56). IL-23 synergizes with IL-6 to promote the differentiation, survival and maintenance of Th17 cells, and IL-23 can amplify the Th17 cell response by inducing the production of proinflammatory cytokines (57). Therefore, IL-23 plays an important role in the expansion and survival of Th17 cells. High levels of IL-23 have been reported in PBMCs from active BD patients (52, 58). These results suggest that the serum IL-17A/F levels parallel the IL-23 levels in patients with active BD, that the active inflammatory state may lead to the differentiation of Th17 cells, and that the IL-17/23 axis has a significant role in mediating inflammatory responses in BD.

IL-21 is a member of the IL-2 family of cytokines and its function is mediated by the IL-21 receptor (59). IL-21 can stimulate the differentiation of Th17 cells; additionally, IL-21 promotes the expansion of effector CD8^+^ T cells in combination with IL-7 or IL-15 (60) and can activate NK cells (61). Furthermore, IL-21 is critical for B cell differentiation into plasma cells and antibody class switching via the induction of Blimp-1 and Bcl-6 and negatively regulates the function of DCs (62, 63). Wang et al. reported that the development of EAU is related to the production of IL-21 and IL-2 by T cells in the retina (64). This study suggested that IL-21 is correlated with autoimmune diseases, including BD.

Maintaining a proper balance between regulatory T (Treg) cells and effector Th17/Th1 cells is important to ensure effective immunity while preventing pathological autoimmunity (65). Recent studies have shown that the Th17/Th1 balance and the Th17/Treg balance are important in the regulation of inflammation in patients with active BD. The ratio of Th17/Th1 is higher in BD patients than in healthy controls (66). Geri et al. demonstrated the key role of IL-21 in regulating Th17 and Treg cells in BD. IL-21 is produced by CD4^+^ T cells and is related to the increase in Th17 cells and the decrease in Treg cells in peripheral blood. This study also suggested the presence of IL-21- and IL-17A-producing T cells within the cerebrospinal fluid (CSF), brain parenchyma inflammatory infiltrates, and intracerebral blood vessels from active BD patients and the involvement of the CNS (67, 68). Th17 and Treg cells are implicated in inflammatory and autoimmune diseases. Th17 cells are involved in pathological induction and proliferation, while Treg cells inhibit autoimmunity and develop tolerance to self-antigens (69). The Th17/Treg balance provides the basis for understanding and regulating the immunological mechanisms of BD. Therefore, BD is dominated by the immune response of Th1 and Th17 cells. Th1 and Th17 cells are related to active inflammation in BD, and the Th1/Th17 balance, Th17/Treg balance and IL-17/23 axis play important roles in inflammatory and pathological responses of BD patients.

### Th22 Cells and Cytokines in Behcet's Disease

Th22 cells are a subset of CD4^+^ effector T cells distinct from the Th1, Th2, and Th17 subtypes. In the presence of IL-6 and TNF-α, activated naïve CD4^+^ T cells differentiate into Th22 cells. Th22 cells mainly secrete IL-22 and TNF-α and express the chemokine receptors CCR4, CCR6, and CCR10 (70). IL-22 is a member of the IL-10 cytokine family, and the IL-22 receptor complex is composed of IL-22R1 and IL-10R2 (71). IL-22 is an inflammatory cytokine that promotes inflammation and is associated with autoimmune diseases. In addition, accumulating evidence suggests that IL-22 plays an important role in the pathogenesis of autoimmune diseases (72). Sugita et al. showed that Th22-type T cell clones could be established from ocular samples obtained from patients with active BD, these clones produced numerous Th22-associated cytokines and overexpressed IL-22, TNF-α, and CCR10. In addition, fresh T cells from patients with BD expressed high levels of Th22-related molecules. However, when fresh T cells from patients with BD were treated with infliximab, the expression of these molecules was very low. Thus, the inhibitory effect of anti-TNF-α therapy on T cell differentiation may protect against severe ocular inflammation in patients with BD (73).

However, other studies have suggested that the IL-22 level in the supernatant of stimulated PBMCs in BD patients with active uveitis was higher than that in patients without uveitis or in normal controls and that the level of IL-22 was associated with the severity of retinal vasculitis and anterior chamber inflammation (74). The increased levels of IL-22 in patients with BD with mucocutaneous lesions might be related to the recurrence of ulcers in the skin and mucosa. The dynamic proinflammatory and anti-inflammatory changes in IL-22 might be associated with the organ involvement and severity of BD (75).

### Treg Cells and Cytokines in Behcet's Disease

Tregs are considered a sublineage of CD4^+^ T cells that have a central role in controlling immune tolerance and maintaining immune homeostasis (76, 77). Treg cells specifically express the transcription factor Foxp3 (forkhead box protein P3) and highly express surface and intracellular markers, including CD25, GITR, and CTLA-4, which can suppress the activation, proliferation and effector functions of a wide range of immune cells (78). However, data on Treg cell populations in BD patients are contradictory. In some studies, the ratio of Treg cells was found to be increased in the peripheral blood and CSF (79), while in other studies, the ratio was found to be decreased (67). Several potential mechanisms for Treg cell suppressive functions have been identified, which are mainly mediated by cell-cell contact, cytokine secretion and metabolic disruption. Treg cells can express immunosuppressive cytokines such as IL-10, IL-35, and TGF-β (80).

IL-10 was originally described as a cytokine secreted by Th2 cells. But further studies have shown that IL-10 can also be produced by innate immune cells (81). The SNP rs1518111 was replicated in Middle Eastern Arab, Greek, British, and Han Chinese samples, and rs1800872 was replicated in Turkish, Korean, and Han Chinese samples (82). IL-10 has especially important anti-inflammatory and immunosuppressive effects, such as inhibiting the function of APCs, inducing the differentiation of Treg cells, and controlling the proliferation of other T cell populations (83). In addition, IL-10 inhibits the apoptosis of B cells, promotes the proliferation of B cells, and stimulates the expression of MHC class II molecules and the cytotoxic activity of NK cells (84). Moreover, there is evidence that IFN-α is an effective treatment for patients with BD (85). Touzot et al. explained that the underlying mechanism in BD was the IFN-α-mediated stimulation of a regulatory Th1 response through the secretion of IL-10. However, the evidence also showed that IFN-α did not directly regulate the Th1/Th17 balance in BD but increased the IL-10/IL-6 ratio, which led to the anti-inflammatory state of memory CD4 T cells (86). Liu et al. showed that IFN-α inhibited the expression of IL-17 and increased the production of IL-10 *in vitro* in PBMCs and CD4 T cells from BD patients and that the inhibitory effect of IFN-α on IL-17 was partly mediated by IL-10 (87). These studies not only suggest that the possible mechanism of IFN-α in the treatment of patients with BD is related to IL-10 but also confirm the anti-inflammatory and immunosuppressive characteristics of IL-10.

IL-35 is the most recently identified member of the IL-12 cytokine family and may be a new target for the treatment of autoimmune and inflammatory diseases. IL-35 has an immunosuppressive effect that is achieved via regulatory T and B cells (88, 89). In contrast to the proinflammatory effect of other cytokines in the IL-12 family, IL-35 inhibits CD4^+^ effector T cells, including Th1 and Th17 cells, by amplifying the Treg cell response and producing IL-10 (88). Recent findings suggest the abnormal expression of IL-35 in inflammatory autoimmune diseases, including EAU. In addition, functional analysis indicates that IL-35 plays a key role in the occurrence and development of autoimmune diseases (90). Sonmez et al. reported that a low level of the Treg cytokine IL-35 was negatively correlated with the number of Th17 and Treg cells. The level of IL-35 in patients with inactive BD and healthy controls was higher than that in patients with active BD, a finding that could be explained by the plasticity between Th17 cells and Treg cells (58).

## The Role of Other Cytokines in Behcet's Disease

### Proinflammatory Cytokines in Behcet's Disease

IL-1, IL-6, and TNF-α are major proinflammatory cytokines in patients with BD. These cytokines have been found in the ocular fluid of patients with BD for more than 20 years and are believed to be the major inflammatory mediators leading to the development of the disease (91). Recent studies have shown that single nucleotide polymorphisms (SNPs) of these cytokines are associated with the onset of BD and that gene polymorphisms are involved in the pathogenesis of BD, which leads to the increased expression of these proinflammatory cytokines (92–94).

IL-6 is clearly a pleiotropic cytokine, which is produced by innate immune cells (59). IL-6 production is tightly negatively regulated, and abnormal excessive production of IL-6 has been found to be related to autoimmune and chronic inflammatory diseases (95). In the absence of substantial inflammation, TGF-β promotes Treg cell differentiation and maintains immune tolerance due to the FoxP3-mediated inhibition of RORγt activity, resulting in the blockade of IL-17 and IL-23 expression. Under inflammatory conditions, IL-1β, IL-6, and IL-21 play a key role in the relationship between Treg and Th17 cells by controlling the FoxP3/RORγt balance (57, 96). IL-1β and IL-6 are responsible for promoting the expansion of differentiated Th17 cells, and the combination of TGF-β and IL-21 is sufficient to induce the differentiation of naïve T cells into Th17 cells (97). The increase in IL-6 in the CSF of patients with neuro-BD has been reported to be associated with long-term prognosis and disease activity and is regarded as a marker of disease activity (98, 99).

TNF-α is a representative proinflammatory cytokine and plays a central role in the induction and maintenance of inflammation in the autoimmune response. In inflammatory diseases, TNF-α is mainly produced by cells of the monocyte/macrophage lineage, but a wide range of cells can produce TNF-α, including T cells, B cells, neutrophils, NK cells, and endothelial cells (100). All known responses to TNF-α are triggered by the binding of TNF-α to one of two distinct receptors, TNFR1 and TNFR2. Therefore, there are two kinds of anti-TNF-α mediators, namely, monoclonal antibodies and soluble receptors, which are characterized by their mechanisms of action (85). Over the past decade, the off-label use of TNF-α antagonists such as infliximab, adalimumab, etanercept and golimumab has improved the treatment of refractory immune-mediated uveitis, especially in BD, and there is sufficient evidence to suggest that TNF-α inhibition is an important development in the treatment of patients with severe and resistant BD (101–103).

### Anti-inflammatory Cytokines in Behcet's Disease

IL-37 was first described as an anti-inflammatory cytokine in autoimmune and inflammatory diseases. IL-37 production can be induced in PBMCs, epithelial cells, DCs and monocytes (104). As a protective mechanism against excessive tissue damage, the production of IL-37 is activated by proinflammatory stimuli, such as IL-1, IL-6, and TNF-α. Therefore, the level of IL-37 may change in autoimmune and inflammatory diseases (105). Recent studies have shown abnormal IL-37 expression in autoimmune diseases, including Behcet's disease, and functional analysis has shown that IL-37 expression is negatively correlated with the development and pathogenesis of BD (106, 107). Bouali et al. indicated that in the serum and PBMC culture supernatants from patients with active BD, the level of IL-37 was decreased, while the levels of IL-1, IL-6, and TNF-α were increased. Corticosteroid treatment of patients with active BD was related to an increase in the IL-37 mRNA and protein levels, suggesting that this treatment may play an immunosuppressive role by regulating the production of IL-37 and reducing the levels of the proinflammatory cytokines IL-1, IL-6, and TNF-α (108).

IL-27 is a heterodimeric cytokine composed of the p28 and EBI3 subunits and belongs to the IL-12 cytokine family with IL-12, IL-23, and IL-35 (109). IL-27 receptors are widely expressed in various types of cells, such as naïve T cells, DCs, NK cells, monocytes, activated B cells, and vascular endothelial cells, and IL-27 is mainly produced by activated antigen-presenting cells (110). IL-27 appears to have two different functions in the immune response: to promote the Th1 immune response and to attenuate the immune/inflammatory response. IL-27 has been shown to have immunosuppressive properties, which can inhibit experimental autoimmune encephalomyelitis (EAE) and EAU by inhibiting the development or proliferation of Th17 cells and inducing the production of IL-10 (111–113). In the absence of IL-27-mediated immunosuppression, the secretion of various inflammatory cytokines is accompanied by severe inflammatory reactions (110). The expression of IL-27 in patients with active BD was reported to be lower than that in normal controls. In addition, recombinant IL-27 inhibited the differentiation of Th17 cells in both BD patients and healthy controls through the interferon regulatory factor 8 (IRF8) pathway (114). The decrease in IL-27 expression was associated with intraocular inflammation in BD, suggesting that IL-27 was involved in the occurrence and development of BD.

## The Latest Treatments for Behcet's Disease (Figure 3)

Despite the traditional options of glucocorticoid and immunosuppressive therapies, the visual outcomes and prognoses of patients with BD have not improved substantially until recently with the advent of biotherapies. IFN-α and TNF-α antagonists have shown good efficacy and are the first-line agents used to improve the prognosis of BD. IFN-α was the first biological agent used to treat BD before the emergence of anti-TNF. Currently, two different types of human recombinant IFN-α (IFN-α-2a and INF-α-2b) are commercially available. IFN-α treatment can achieve long-lasting remission of BD, and no drugs are used during remission (115, 116). A retrospective study from France of long-term efficacy evaluation IFN-α in the treatment of severe uveitis showed that 58% of BD patients were able to discontinue treatment and 81% of them had no recurrence within 5 years of IFN-α treatment. During the study period, the visual acuity improved or stabilized in 89% of the eyes (117). TNF-α antagonists are divided into monoclonal antibody (infliximab, adalimumab, and golimumab) and soluble receptor (etanercept) classes according to their different mechanisms of action. Anti-TNF therapy has been reported to be rapidly effective in inducing and maintaining remission (101, 118). A retrospective multicenter study in South Korea showed that routine drug treatment was ineffective in 28 patients with intestinal BD, and the clinical effective rate was 64.8% after 4 weeks of infliximab treatment (119). Further randomized prospective trials are necessary to confirm these findings, although there is evidence that TNF-α antagonists therapy for intestinal BD is effective (120). More recently, accumulating studies have reported the use of agents that target interleukins and their receptors, such as IL-1 blockers (anakinra and canakinumab), an IL-6 blocker (tocilizumab), an IL-17 blocker (secukinumab), and a monoclonal antibody targeting IL-12/IL-23 (ustekinumab) (121, 122). In a recent phase 2 study, an oral phosphodiesterase 4 inhibitor, apremilast, was found to be very effective in inhibiting oral ulcers (123). For the management of BD, recent studies have shown that apremilast, anakinra, and ustekinumab are effective for the treatment of refractory mucocutaneous involvement. New data from a series of cases confirmed the effectiveness and safety of TNF-α inhibitors in the treatment of refractory disease in major organs. For refractory ocular disease, a long-term follow-up study also confirmed the efficacy and safety of IFN-α. IL-1 inhibitors and tocilizumab appear to be alternatives for patients with refractory ocular involvement (124).

**Figure 3:**
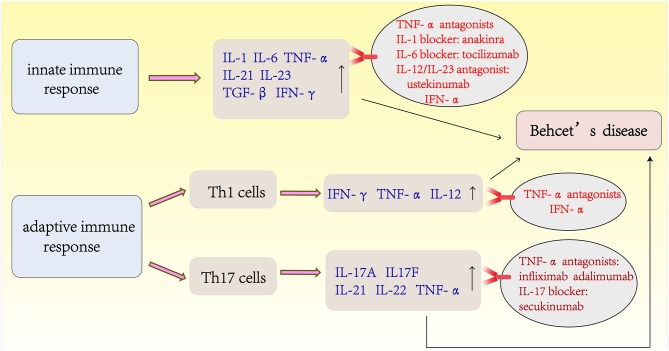
The latest treatments of Behcet's disease is based on the immunopathogenesis of BD.

## Conclusion

Although the etiology of BD is still unclear, advances in genetics and immunology have led to a better understanding of the immunopathogenesis of BD. Infection-related trigger factors are believed to be involved in the development of BD in patients with a genetic predisposition. The HLA-B^*^51 allele and variants in IL-10 and at the IL-23–IL-12RB2 locus are the known genetic factors most closely associated with BD. Trigger factors include bacterial infections, viral infections and abnormal autoantigens (such as HSPs, S antigen, and IRBP). Subsequently, the innate and adaptive immune systems are activated by these trigger factors, resulting in the production of numerous cytokines and chemokines to counteract the antigens and autoantigens. In the innate immune system, NK cells, γδ T cells and neutrophils are the primary cells involved in the pathogenesis of BD. NK cells not only play a cytotoxic role in infected cells and tumor cells but also regulate the function of other immune cells, including DCs and T cells, through the secretion of cytokines. BD is characterized by venous thrombosis, aneurysms and occlusions. Histopathological analysis has shown that arteries and veins are infiltrated by neutrophils and lymphocytes, which results in vascular endothelial dysfunction. Endothelial dysfunction and neutrophil vascular inflammation are key factors mediating thrombosis in patients with BD. CD4^+^ T cells, including Th1, Th2, Th17, Th22, and Treg cells, and related cytokines in the adaptive immune system play key roles in the pathogenesis of BD, and cytokines undoubtedly play a vital role in the initiation and perpetuation of BD. A better understanding of the mechanisms associated with inflammatory responses and adaptive immune system regulation in BD promotes the development of biotherapies. The clinical application of these biologic agents and their good therapeutic effects in BD are based on our in-depth understanding of the immunopathogenesis of BD. Although biologics to treat BD are expensive and most are still in clinical trials, the treatment effect in patients with refractory BD is worth anticipating.

## Author Contributions

BT carried out the primary literature search, wrote, and revised the manuscript. GS initiated the concept and supervised the writing and revision of the manuscript. XL and JX were involved in the preparation and revision of the manuscript. All authors read and approved the final manuscript.

### Conflict of Interest Statement

The authors declare that the research was conducted in the absence of any commercial or financial relationships that could be construed as a potential conflict of interest.
